# Establishment of a triplex TaqMan quantitative real-time PCR assay for simultaneous detection of *Cymbidium mosaic virus*, *Odontoglossum ringspot virus* and *Cymbidium ringspot virus*

**DOI:** 10.3389/fmicb.2023.1129259

**Published:** 2023-05-19

**Authors:** Aiqing Sun, Lihua Wang, Yiping Zhang, Xiumei Yang, Yi Wei, Dong Yang, Wenhan Li, Xuewei Wu

**Affiliations:** ^1^Flower Research Institute, Yunnan Agriculture Academy of Science Kunming, Kunming, Yunnan, China; ^2^Yunnan University, Kunming, Yunnan, China

**Keywords:** triplex qRT-PCR, CymMV, ORSV, CymRSV, viruses detection, orchids

## Abstract

Orchids are significant ornamental plants whose viral infection results in substantial economic damage. *Cymbidium mosaic virus* (CymMV), *Odontoglossum ringspot virus* (ORSV), and *Cymbidium ringspot virus* (CymRSV) represent three important and prevalent orchid viruses. The detection system proposed in this study uses a triplex TaqMan quantitative real-time PCR assay to identify CymMV, ORSV, and CymRSV in a simultaneous manner. We designed specific primers and probes for CymMV, ORSV, and CymRSV, with amplified sequences of 156 bp, 148 bp, and 145 bp, respectively. The minimum detection limit of the triplex qRT-PCR assay for CymMV and CymRSV was 1 copy/assay, and the minimum detection limit was 10 copies/assay for ORSV. The minimum stable detection limits for CymMV, ORSV, and CymRSV were 10, 10^2^, and 10^2^ copies/assay, respectively. Therefore, this system exhibited higher sensitivity (approximately 10 to 10^4^-fold) than RT–PCR. The intra-and interassay CVs of Cq values are less than 0.55 and 0.95%, respectively, indicating that the triplex assay is highly reliable and accurate. In addition, 66 samples from five different orchid genera were analyzed using the established assay and gene chip. The detection results demonstrated that the triplex probe qRT–PCR demonstrated higher sensitivity than the gene chip, indicating that the triplex real-time PCR assay could be used for the detection of field samples. Our findings suggest that the triplex real-time RT–PCR detection system represents a rapid, simple, and accurate tool for detecting CymMV, ORSV, and CymRSV on orchids.

## 1. Introduction

Orchids are significant ornamental plants from the Orchidaceae family, representing economically popular ornamental cut-flowers and potted floricultural crops worldwide (Ali R. N. et al., 2014; Yusop et al., 2022). However, more than 50 viruses are known to infect orchids globally, and *cymbidium mosaic virus* (CymMV) and *odontoglossum ringspot virus* (ORSV) are the two most prevalent and economically essential orchid viruses (Wong et al., 1994; Pant et al., 2020; Jia et al., 2004) that achieved a worldwide distribution, infecting numerous commercially significant orchid genera (Zettler et al., 1990; Jia et al. 2004). *Cymbidium ringspot virus* (CymRSV) is a virus of serious concern that infects orchids, primarily in Europe, North America, South America, and other locations (Medzihradszky et al., 2019). CymMV, ORSV, and CymRSV have all been listed as orchids quarantine viruses in China (Ming et al., 2010). As international requirements for virus detection have increased in recent years (Khentry et al., 2006; Chen and Jiang, 2017), the absence of certain quarantine viruses is a prerequisite for the smooth customs clearance of national import and export trade. However, as orchid trade and germplasm exchange have recently increased worldwide, the incidence of orchid virus diseases has shown a tendency to spread. Most orchids are infected with one or a combination of two viruses, and coinfection with a combination of these three viruses has also been reported (Peng, 2015). When two or more viruses combine to cause an infection, the symptoms are severe, and the degree of damage has a characteristic of 1 + 1 > 2, which significantly impacts the ornamental value and commodity value. Nevertheless, infected orchids occasionally exhibit only mild symptoms or no symptoms at all in the early stages and are frequently unable to be identified by simply observing symptoms (Koh et al., 2014). Although these plants may evade identification, they are still infectious (Kim and Choi, 2015). Furthermore, there is currently no effective agricultural chemical for virus-carrying plants, so it is crucial to conduct efficient virus detection on plants as early as possible in order to detect and isolate infected plants as early as possible, which is crucial for enhancing product quality and minimizing field losses. Therefore, a rapid, precise, and sensitive method is required to detect and identify these viruses at the species level.

The current methods for detecting these viruses include virus isolation and identification, serological, and polymerase chain reaction methods (Schaad et al., 2003; Digangi et al., 2011; Streck et al., 2013; Kim et al., 2020). Rapid detection technology is an active area of study. Among these technologies, ELISA can be used for large-scale detection, whereas molecular approaches are primarily employed for species-specific detection (Liang et al., 2015; Zhang et al., 2016). Occasionally, serological diagnosis can yield a false-positive result, and virus isolation has not been widely used in clinical diagnosis, given its high costs and time-consuming methodology (Digangi et al., 2011). The PCR method has been widely used in the diagnosis of various pathogens due to its specificity, sensitivity, and efficiency (Elia et al., 2006; Decaro et al., 2008). Compared to conventional detection methods, PCR-based technologies can significantly reduce the time needed for diagnosis. PCR-based technologies can significantly reduce the time required for diagnosis compared to conventional detection methods (Chamberlain and Chamberlain, 1994; Ali M. E. et al., 2014). However, conventional PCR is unsuitable for the quantitative analysis of plant pathogens (Sanzani et al., 2013). In contrast, quantitative real-time PCR (qRT–PCR) is a versatile advanced technology that allows one to distinguish the target pathogen simply, quickly and economically with excellent repeatability (Cao et al., 2022). Additionally, qRT–PCR can distinguish between two or more closely related organisms. The use of probes with different fluorescent dyes enables the detection of several target pathogen DNAs or RNAs in a single reaction (multiplex-PCR), and this technique has been widely utilized in the field of scientific research (Espy et al., 2006; Bustin et al., 2009; Cao et al., 2022).

Although several PCR methods have been reported for detecting CymMV, ORSV, and CymRSV, the majority of them have low sensitivity or are tedious to perform. Moreover, there is currently no method that can simultaneously detect these three viruses with speed and with high sensitivity (Ali R. N. et al., 2014; Kim and Choi, 2015; Zheng et al., 2018). In this study, a TaqMan triplex qRT–PCR assay (triplex assay) was developed for the simultaneous detection of CymMV, ORSV, and CymRSV. This method demonstrated high sensitivity, specificity, and repeatability in distinguishing these viruses.

## 2. Materials and methods

### 2.1. Orchid material

Samples infected with CymMV and ORSV single virus or both viruses and healthy *Phalaenopsis amabilis* were taken from the Orchids Plant in Kaiyuan, Yunnan Province, China. In our laboratory, single virus samples infected with CMV, TMV, CarMV, or LMoV were stored. Samples infected with CymRSV were purchased from Agdia (United States) and confirmed by RT-PCR. The orchids cultivars included “Large chili pepper”, “Asahi”, “Greenbear”, and others. From the Kaiyuan orchids factory, we also obtained *Dendrobium nobile*, *Zygopetalum*, *Haraella retrocalla*, and *Oncidium hybridum* samples.

### 2.2. Primer and probe design

Multiple coat protein sequences of CymMV were downloaded from the NCBI. Based on DNAMAN comparison and according to RT–qPCR primer design requirements, three pairs of specific primers and probes were designed using Primer 5.0 software in highly conserved regions of the virus sequences. Primer-blast comparisons were performed in NCBI to ensure primer specificity. The specific primers were screened through RT-PCR, followed by agarose gel electrophoresis, primers that can produce bright and single amplicons were selected for further screening utilizing the TB Green dye method. For triplex taqman qRT-PCR fluorescence detection, primers given rise to a single dissolution curve and high amplification efficiency in the TB Green dye method were utilized. The method for selecting primers for ORSV and CymRSV was identical to that for CymMV. The three probes were labeled with FAM/BHQ1 (CymMV), HEX/BHQ1 (ORSV), and Cy5/BHQ3 (CymRSV) at their 5′ and 3′ terminals. The primers and probes were synthesized by Sangon Biotech (Shanghai, China), and the details of these oligos are shown in Table 1.

**Table 1:** Primers and probes.

Name	Sequence (5′-3′)	Fragment length (bp)
CymMV-F	CTGATGCTGGCCACTAACGA	156
CymMV-R	CACGTTCACGGTCAGTAGGG	
CymMV-Probe	FAM-CCGCCAACTGGGCCAAGGCT-BHQ1	
ORSV-F	TTGACCAGTAGGTTCCCTGC	148
ORSV-R	TAGTTGTCGGATTCTGCGGAT	
ORSV-Probe	HEX-TGGTTACTTCAGAGTTTATCGCTATG-BHQ1	
CymRSV-F	CGCAGTGGGTGACTTATT	145
CymRSV-R	CGTCGTGGCTGTGGTAG	
CymRSV-Probe	Cy5-CACAGTAACCTTCTACGAACCGCAACCG-BHQ3	

### 2.3. Nucleic acid extraction and standard template plasmid preparation

The total RNA was extracted from three samples only infected with a single virus of CymMV, ORSV, or CymRSV using a Mini BEST Plant RNA Extraction Kit (Takara, China), quality and quantity were assessed using a Nanodrop2000 spectrophotometer (Thermo, United States). Subsequently, cDNA was synthesized employing the PrimeScript RT Reagent Kit (Takara, China) with gDNA Eraser to perform the reverse transcription reaction. The reverse transcription reaction system and the reaction procedure are described as follows: 5 × g DNA Eraser Buffer 2.0 μL, gDNA Eraser 1.0 μL, total RNA 7.0 μL; 42°C 2 min, 4°C insulation; PrimeScript RT Enzyme Mix I 1.0 μL, RT Primer Mix 4.0 μL, 5 × PrimeScript Buffer 2(for Real Time) 4.0 μL, RNase Free ddH_2_O 1.0 μL; 37°C 15 min, 85°C 5 s, 4°C insulation.

Using the obtained three cDNA as templates, the annealing temperature of CymMV, ORSV, and CymRSV were optimized based on the base sequence of primers, whereas CymMV and ORSV were optimized over different Tm ranged from 55°C to 60°C at 1°C interval, CymRSV were optimized over different Tm ranged from 52°C to 57°C at 1°C interval. The PCR products were sent to Sangon Biotech (Shanghai, China) for sequencing, and the results showed 100% identity to the target sequence. The PCR products were then purified utilizing a DNA Gel Extraction Kit (Takara, China). Retrieved purified DNA fragments were cloned into the pMD19-T vector (Takara, China) to construct three plasmids as positive controls and templates named pMD19T-CymMV, pMD19T-ORSV, and pMD19T-CymRSV. Positive plasmid standards for the three viruses were sequenced to ensure sequence accuracy. The positive plasmids were used to establish standard curves. According to the absorbance measurement, the concentration of plasmids was calculated, and the calculation used to determine the copy number of the plasmid in a previous study was used (Yao et al., 2019). The standard plasmid pMD19T-CymMV, pMD19T-ORSV, and pMD19T-CymRSV were diluted to 10^9^ copies/assay and then mixed (10^9^ copies/assay). The mixed plasmids were subsequently diluted to 10^9^ copies/assay to 1 copy/assay at a 10-fold ratio.

### 2.4. Experimental design and qRT–PCR

To obtain a more sensitive, stable, and efficient qRT–PCR method, the annealing temperature, primer concentration, and probe concentration were carefully optimized for each target gene. The experimental conditions with highest fluorescence signal and the lowest cycle threshold (Cq) value were determined to be the optimal reaction conditions. Using optimized three cDNA (10 < Cq < 35) as templates, CymMV-F, CymMV-R, and CymMV-Probe; ORSV-F, ORSV-R, and ORSV-Probe. CymRSV-F, CymRSV-R, and CymRSV-Probe were used for the real-time quantitative PCR amplification system and program screening. The annealing temperature (58°C, 60°C, and 62°C), primer concentration (1 μM, 10 μM, 20 μM, and 50 μM) and probe concentration (1 μM, 10 μM, 20 μM, and 50 μM) were optimized using the matrix method. The optimal annealing temperature and the concentration of primers and probes were selected for each virus separately. Then, the optimization of triple detection system was further optimized to identify the most optimal conditions for triple detection by further optimizing the proportion of primers and probes added for the three viruses and the annealing temperature based on the optimal detection system for a single virus.

The mixed plasmid containing 10^9^ to 10^2^ copies/assay was used as the template, and ddH_2_O served as the negative control. These samples were amplified using triplex qRT-PCR based on the optimized reaction systems. When established the dynamics and standard curves, the amplification efficiency (*E*) and correlation coefficient (*R*^2^) were used as parameters to evaluate the triplex assay.

### 2.5. Validation of the qRT–PCR assay

The specificity of the established qRT–PCR assay was confirmed using *Cucumber mosaic virus* (CMV), *Tobacco mosaic virus* (TMV), *Carnation mottle virus* (CarMV), and *Lily mottle virus* (LMoV). CMV, CarMV, and CymRSV are spheroids viruses of an equiaxed icosahedron, and CarMV and CymRSV belong to Tombusviridae. Both TMV and ORSV are rhabditiform viruses, and both belong to Tobamovirus. LMoV belongs to Potyvirus, CymMV belongs to Potexvirus, whereas both LMoV and CymMV are linear viruses. The above five viruses extracted RNA from the leaves of infected plants, the reverse transcription for cDNA was verified by PCR, then cDNA was used for fluorescence quantitative specificity detection. In order to validate the sensitivity of the method, a mixed 10^9^ copies/assay to 1 copy/assay plasmid was used as the template and subjected to the sensitivity of the triplex assay. The repeatability of the method was tested using plasmids as templates at 10^4^, 10^5^, 10^6^, 10^7^, and 10^8^ copies/assay, respectively. For the intra-assay repeatability test, each independent experiment was conducted in triplicate, and for the inter-assay repeatability test, triplicate runs were conducted.

### 2.6. RT–PCR

The reaction system and reaction conditions of the RT-PCR procedure for sequence amplification and sensitivity verification are as follows (according to the PCR amplification kit instructions). RT–PCR was performed with a standard protocol in a 25.0 μL reaction volume on a PCR cycle (Bio-Rad, United States). Reaction mixture components included 2.5 μL 10 × Ex Taq Buffer, 2.5 μL dNTP Mixture, 0.25 μL Ex Taq, 0.5 μL 20 μM Primer F, 0.5 μL 20 μM Primer R, 2.5 μL cDNA, and 16.25 μL RNase-free ddH_2_O. The cycle program was as follows: 95°C for 5 min, followed by 35 cycles of 95°C for 30 s, Tm for 30 s (Tm (CymMV) = 58°C, Tm (ORSV) = 58°C, Tm (CymRSV) = 55°C), and 72°C for 1 min, and a final step of 10 min at 72°C. The PCR products were detected by 1.0% agarose gel electrophoresis. Three standard plasmids were used as positive controls, and ddH_2_O served as the negative control.

### 2.7. Statistical analysis

The CFX96 Real-Time PCR system (Bio-Rad, Germany) was utilized for data generation and data collection. Using Microsoft Excel 2017, data management, analysis, and graphic generation were performed. The intra-and interassay variations were calculated from the Cq values, and the results were expressed as the mean value, standard deviation (SD) and coefficient of variation (CV).

### 2.8. Screening a variety of orchids of the triple qRT–PCR

Sixty-six samples, including 16 *Phalaenopsis*, 17 *Zygopetalum*, 17 *Oncidium hybridum*, and 16 *Dendrobium* were collected from the Orchids Plant in Kaiyuan, Yunnan Province, China. The triplex qRT-PCR was used to screen the samples for CymMV, ORSV, and CymRSV. Additionally, ddH_2_O was used as the negative control, and the three virus mixed plasmids (10^7^ copies/assay) as the positive control.

## 3. Results

### 3.1. Optimization and establishment of the triplex assay

Optimizing the single virus detection system of CymMV, ORSV, and CymRSV, the experimental conditions with the highest amplification efficiency (E) and correlation coefficient (R^2^) value were determined to be the optimal reaction conditions. We obtained the optimal reaction conditions for CymMV with a primer concentration of 20 μΜ, a probe concentration of 10 μΜ and an annealing temperature of 58°C. The optimal reaction conditions for ORSV included a primer concentration of 10 μΜ, a probe concentration of 10 μΜ and an annealing temperature of 60°C. The optimum system for CymRSV included a primer concentration of 20 μΜ, a probe concentration of 10 μΜ for the probe, and an annealing temperature of 60°C. To ensure adequate efficiency of the TaqMan primer-probe sets in the presence of other oligonucleotides and fluorogenic dyes, we optimized the amount of primers and probes as well as the annealing temperature of three viruses (Supplementary Figure S1). The triplex assay was performed in a final reaction volume of 25.0 μL on a CFX96 Real-Time PCR system (Bio-Rad, United States). The reaction mixture included 12.5 μL of Premix Ex Taq (2×), 2.0 μL of template, 0.5 μL of the CymMV primer (20 μM), 0.6 μL of the ORSV primer (10 μM), 0.6 μL of the CymRSV primer (20 μM), 1.0 μL of CymMV probe (10 μM), 1.2 μL of ORSV probe (10 μM), 1.2 μL of CymRSV probe (10 μM), and 3.7 μL of RNase-free ddH_2_O. The thermocycling conditions included 95°C for 30 s followed by 40 cycles of 95°C for 5 s and 59°C for 30 s.

### 3.2. Dynamic curve and standard curve of triplex qRT–PCR

**Figure 1:**
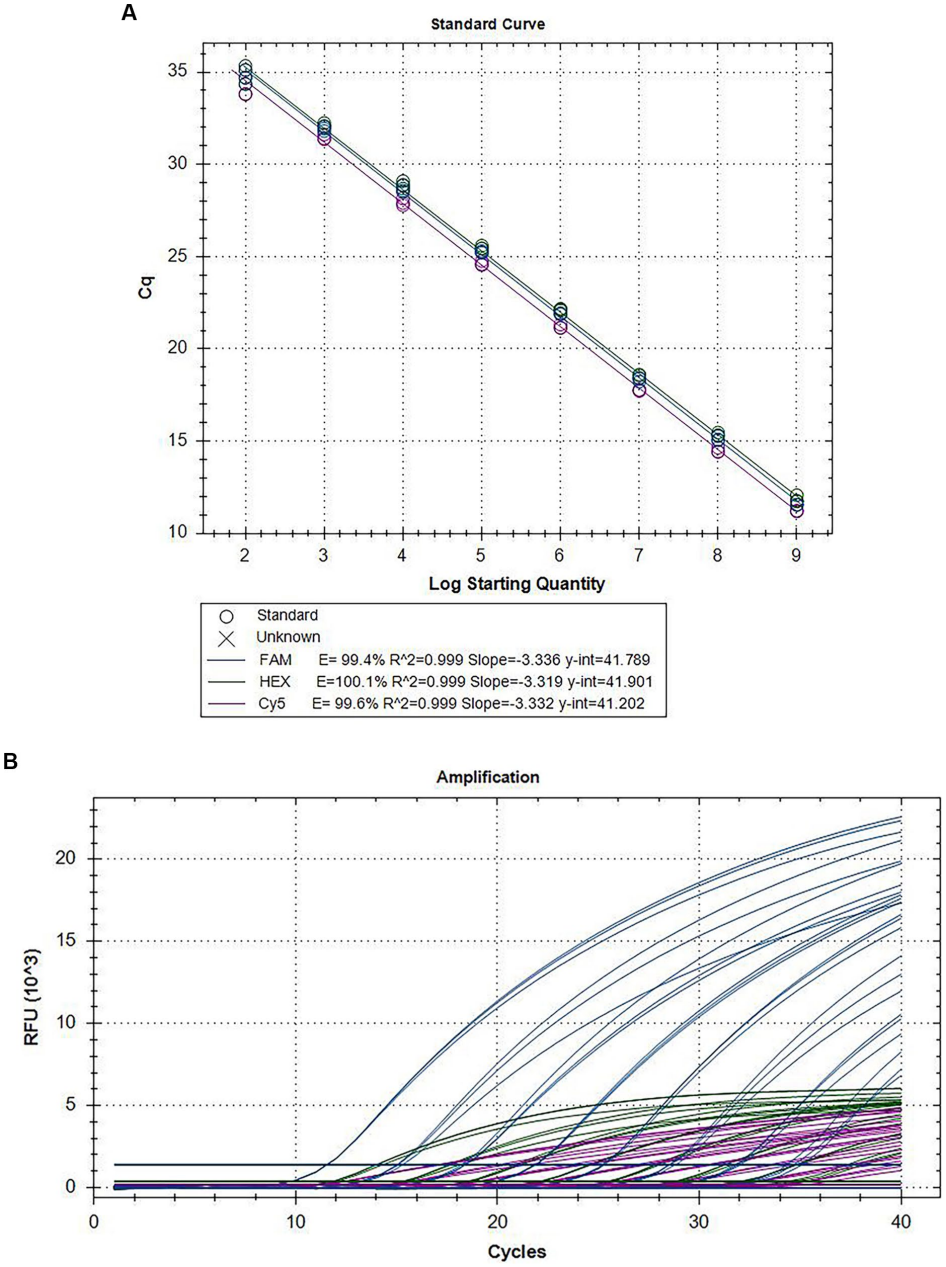
Amplification curves and standard curves of the triplex assay. The mixed plasmids of 10^9^ copies/assay to 10^2^ copies/assay plasmid was used as the template for qRT–PCR. The standard curve **(A)** and the amplification curve **(B)** of the triplex assay was generated by plotting the Cq values (X-axis) against the logarithm of copy numbers of plasmids (Y-axis).

The mixed plasmids were diluted 10-fold to 10^9^ to 10^2^ copies/assay concentration after mixing the three recombinant standard plasmids of CymMV, ORSV, and CymRSV were mixed equally. Based on the optimized reaction systems for the recombinant standard plasmids, both standard and dynamic curves (Figure 1; Supplementary Figure S2) were obtained with these plasmids. The results demonstrated good linearity for CymMV (*R*^2^ = 0.999, *E* = 99.4%), ORSV (*R*^2^ = 0.999, *E* = 100.1%), and CymRSV (*R*^2^ = 0.999, *E* = 99.6%).

### 3.3. Specificity test

**Figure 2:**
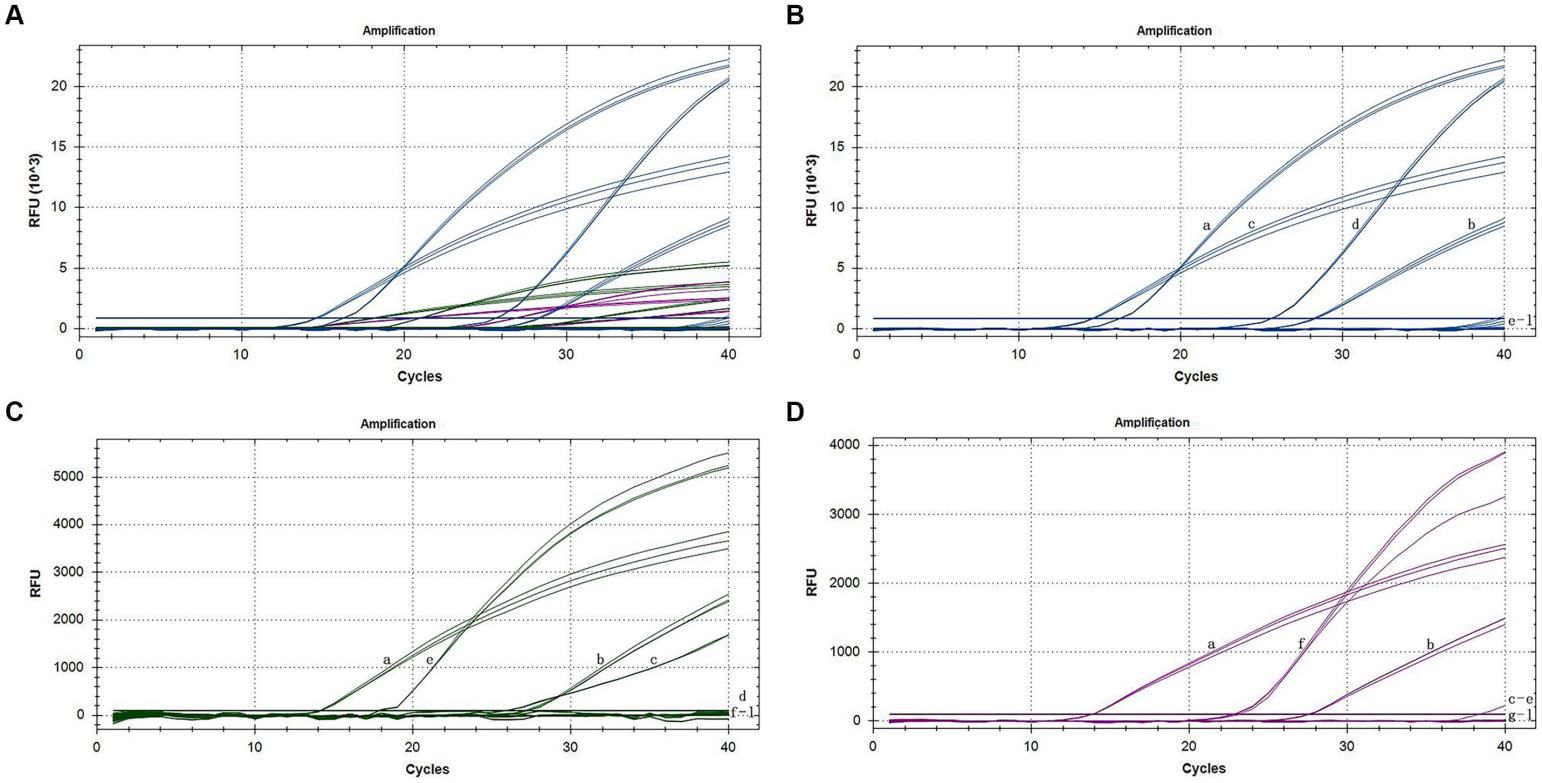
Specific amplification curves of the triplex assay **(A)**. Specific amplification curves of the triplex assay for the detection of FAM channel (CymMV) **(B)**, HEX channel (ORSV) **(C)**, and Cy5 channel (CymRSV) **(D)**. Among them, a: positive sample with CymMV, ORSV and CymRSV; b: sample with CymMV, ORSV and CymRSV; c: sample with CymMV and ORSV; d: sample with CymMV; e: sample with ORSV; f: sample with CymRSV; g–j: samples with CMV, TMV, CarMV, LMoV, respectively; k: negative sample; l: H_2_O.

After adding the various virus isolates to the reactions individually, we obtained a signal only from the specific reporting dye when testing cDNA. This finding demonstrated that each primer-probe set amplifies cDNA from its target species but not from the other two species. Four nontarget viruses (CMV, TMV, CarMV, and LMoV) and cDNA extracted from healthy individuals were not amplified (Figure 2). This finding indicates the high specificity of our primer-probe sets in a triplex assay. When one or two different cDNA samples of orchids were tested together, all three TaqMan primer-probe sets maintained similar specificity and showed discrimination between the particular pathogens present in the triplex assay.

### 3.4. Sensitivity of triplex probe qRT–PCR assay

**Figure 3:**
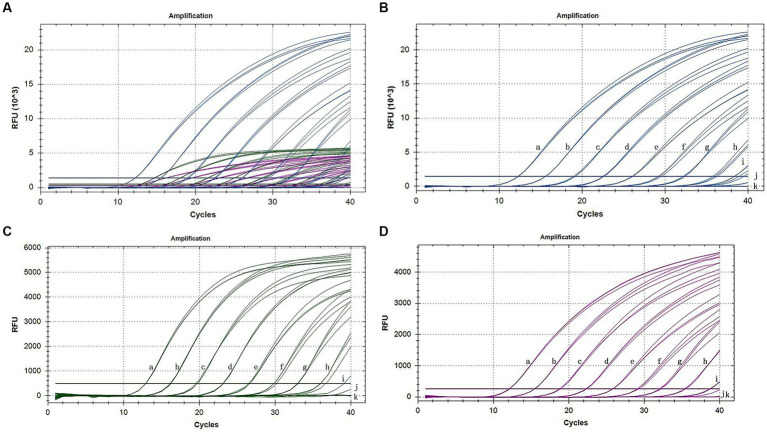
Sensitivity amplification curves of the triplex assay **(A)**. Sensitivity amplification curves of the triplex assay for the detection of FAM channel (CymMV) **(B)**, HEX channel (ORSV) **(C)**, and Cy5 channel (CymRSV) **(D)**. The mixed 10^9^ copies/assay to 1 copy/assay plasmid was used as the sensitivity tests templates.

**Figure 4:**
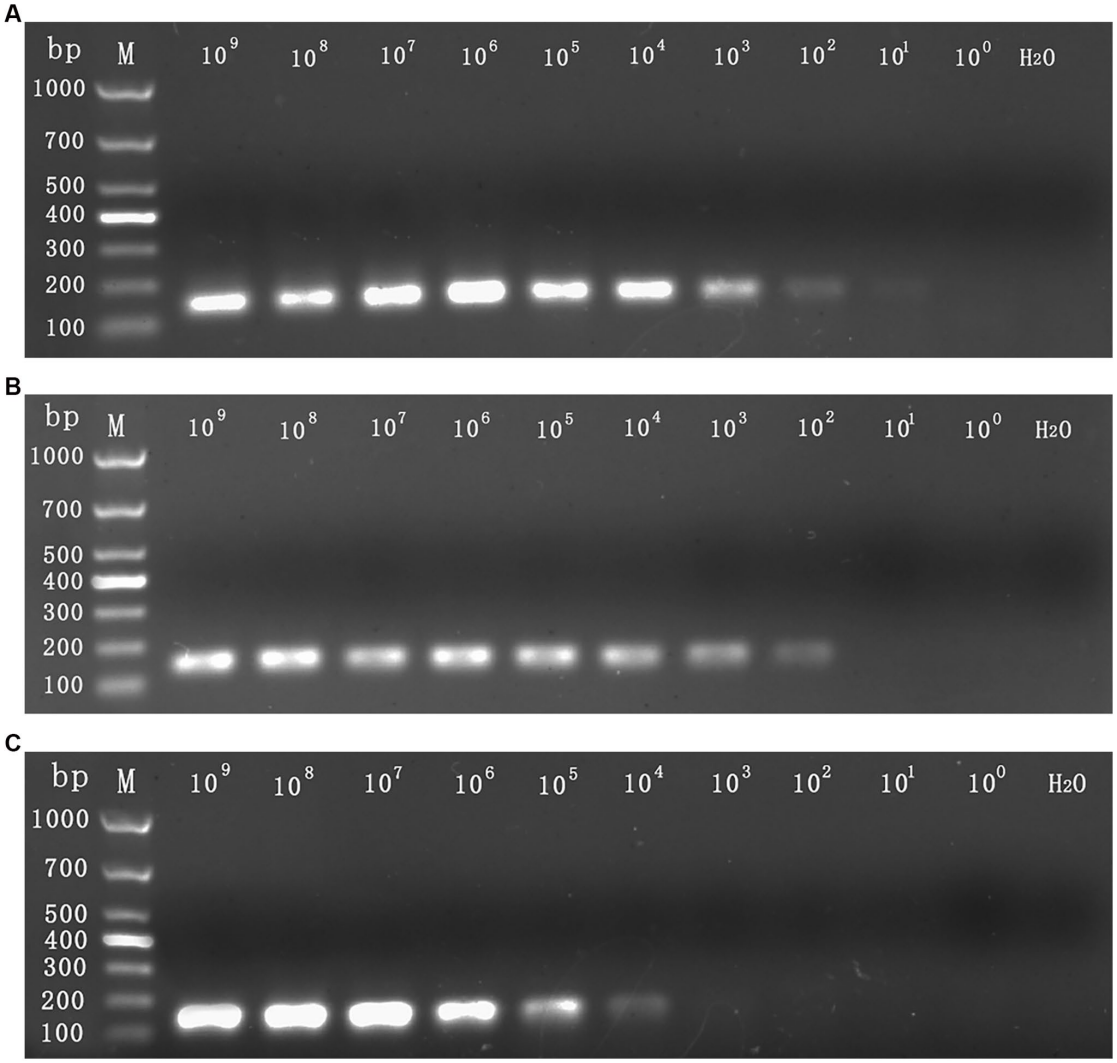
The sensitivity detection limit of RT–PCR. The mixed 10^9^ copies/assay to 1 copy/assay plasmid was used as the sensitivity tests templates. The detection limit for CymMV **(A)** and ORSV **(B)** was 10^2^ copies/assay, the detection limit for CymRSV **(C)** was 10^4^ copies/assay.

The sensitivity assessment of three mixed plasmids (from 10^9^ copies/assay to 1 copy/assay) revealed that the lowest detection limit for CymMV, ORSV, and CymRSV was 1 copy of each. The minimum stable detection limits for CymMV, ORSV, and CymRSV were 10, 10^2^, and 10^2^ copies/assay, respectively (Figure 3). The lowest detection limit of single qRT–PCR sensitivity for the three viruses was 1 copy/assay, and the minimum stable detection limit was 10 copies/assay. The detection limits of RT–PCR were 10 copies/assay, 10^2^ copies/assay, as well as 10^4^ copies/assay for CymMV, ORSV, and CymRSV, respectively (Figure 4). The analytical sensitivity of the developed triplex probe qRT–PCR was increased from 10-fold to 10^4^-fold compared with RT–PCR.

### 3.5. Repeatability of triplex probe qRT–PCR assay

In this study, five different concentrations of plasmids were used for the repeatability test of the established qRT–PCR assay (Table 2). The intra-and interassay CVs of Cq values are less than 0.55 and 0.95%, respectively, indicating that the triplex assay is highly reliable and accurate.

**Table 2:** Repeatability of the triplex probe qRT–PCR assay.

	Number of DNA copies (copies/assay)	Inter-assay			Intra-assay		
		Mean	SD	CV (%)	Mean	SD	CV (%)
CymMV	10^4^	28.65	0.176	0.61	28.76	0.09	0.31
	10^5^	25.25	0.054	0.21	25.32	0.05	0.19
	10^6^	21.90	0.031	0.14	21.92	0.05	0.23
	10^7^	18.35	0.103	0.56	18.49	0.06	0.33
	10^8^	15.15	0.140	0.92	15.19	0.08	0.51
ORSV	10^4^	28.89	0.204	0.71	28.95	0.09	0.31
	10^5^	25.49	0.090	0.35	25.45	0.07	0.29
	10^6^	22.13	0.051	0.23	22.09	0.06	0.28
	10^7^	18.53	0.096	0.52	18.66	0.09	0.46
	10^8^	15.36	0.100	0.65	15.35	0.05	0.32
CymRSV	10^4^	27.95	0.193	0.69	28.22	0.09	0.31
	10^5^	24.64	0.119	0.48	24.86	0.07	0.30
	10^6^	21.21	0.102	0.48	21.37	0.07	0.33
	10^7^	17.76	0.035	0.20	18.04	0.03	0.15
	10^8^	14.51	0.124	0.86	14.69	0.06	0.43

### 3.6. Screening a variety of orchids to evaluate the simultaneous virus detection system

The triplex qRT–PCR assay successfully detected CymMV, ORSV, and CymRSV infections. A total of 66 samples of 16 *Phalaenopsis*, 17 *Zygopetalum*, 17 *Oncidium hybridum,* and 16 *Dendrobium* were collected, and their viral status was examined using the developed triplex assay. The results demonstrated that the positive control showed amplification of all three viruses, and the negative control showed no amplification curves. The results of the sample testing revealed that 30 samples were infected with CymMV, ORSV, or both; however, no plants were tested for CymRSV. The positive rates for CymMV, ORSV, and CymRSV were 30.30% (20/66), 31.82% (21/66), and 0% (0/66), respectively. The 66 samples were also tested by gene chip, and the test results showed that the positive rate of CymMV, ORSV, and CymRSV was 19.70% (13/66), 12.12% (8/66) and 0% (0/66), respectively. According to the results of the two methods, 13/8 samples were positive for CymMV/ORSV by gene chip, among the 53/58 samples detected negative by the gene chip, 7/13 samples detected positive by the triplex assay we established, and 46/45 samples remained negative. No samples were tested for CymRSV infection using either method. The results demonstrated that the triple real-time quantitative PCR system had a higher detection rate than gene chip detection.

## 4. Discussion

Recent years have witnessed an increase in the use of quantitative real-time PCR technology based on TaqMan probes for the uniplex and multiplex detection of viruses and pathogens in plants and animals.

### 4.1. Uniplex qRT–PCR

The sensitivity of quantitative real-time PCR detection of *grapevine fanleaf virus* (GFLV) is 100-fold that of RT-PCR at a detection limit of 10^−5^ ng/μL for cDNA (Zhou et al., 2016). Quantitative real-time PCR detection of *sweet potato latent virus* (SPLV) demonstrated that it can detect the positive plasmid at a concentration of 7 × 10^1^ copies/μL, and the sensitivity is 100-fold that of RT–PCR (He et al., 2020). Similarly, the qRT-PCR detection of *cucumis melo endornavirus* (CmEV) showed that it can detect the positive plasmid at a concentration of 1.8 × 10^3^ copies/μL, and the sensitivity is 100-fold that of RT–PCR (Zhao et al., 2022). Our uniplex qRT–PCR of CymMV, ORSV, and CymRSV revealed its ability to detect the positive plasmid at a concentration of 1 copy/assay of the virus, and the sensitivity is 10^2^ to 10^4^ fold that of RT–PCR.

### 4.2. Optimization of the virus detection system

Hosseini et al. (2021) focused on the detection of quadruplex real-time PCR of *Diaporthe longicolla*, *D. caulivora*, *D. eres*, and *D. novem*, and they reported uniplexes, duplexes, and quadruplex assays for optimization to ensure that each primer-probe exhibits good amplification efficiency. Geng et al. (2020) focused on the detection of triplex real-time PCR of *bovine parvovirus*, *bovine coronavirus*, and *bovine parainfluenza virus*, and they reported that an annealing temperature of 41.7–52°C was optimal for their triple qRT–PCR assay. Ali R. N. et al. (2014) focused on the detection of triple RT–PCR of CymMV, ORSV, and *orchid fleck virus* (OFV), and they optimized the annealing temperature and primer concentration of RT–PCR to describe the most appropriate reaction conditions. Liang et al. (2017) focused on the detection of CymMV using SYBR Green, optimizing the primer concentration, probe concentration, and buffer concentration SYBR Premix Ex TaqII (2×). Consistent with the current research, Cao et al. (2022) demonstrated that the primer concentration, probe concentration, and annealing temperature were optimized for reaction conditions.

### 4.3. Establishment of the virus detection system

Hosseini et al. (2021) reported a standard curve established from 5 dilution gradients (19.4 ng–1.94 pg) with an *R*^2^ of 0.994–0.999 and an *E* value of 87.8–95.6%. Geng et al. (2020) reported a standard curve that was established from 7 dilution gradients (2 × 10^8^ copies/μL to 2 × 10^2^ copies/μL). Cao et al. (2022) reported a standard curve generated from 7 dilution gradients (5 × 10^7^ copies/assay to 5 × 10^1^ copies/assay) with an *R*^2^ of 0.996–0.999 and an *E* value of 87.8–95.6%. Liang et al. (2017) reported a standard curve established from 7 dilution gradients (10^9^ copies/μL to 10^3^ copies/μL) with an *R*^2^ of 0.998 and an *E* value of 100%. According to our research, a standard curve was derived from eight dilution gradients (10^9^ copies/assay to 10^2^ copies/assay) with an *R*^2^ of 0.999 and an *E* value of 99.4–100.1%.

### 4.4. Specificity of the virus detection system

The quadruplex-specific detection system described by Hosseini et al. (2021) used each pathogen’s primer and probe to detect the other three pathogens. The triple one-step multiplex RT–PCR specific detection system described by Thonur et al. (2012) is consistent with that reported by Hosseini et al. The triplex-specific detection system described by Geng et al. (2020) used six viruses commonly found in infected animals. The results indicate that these six viruses have no amplification curve, whereas the viruses in the triple test exhibit an amplification curve. According to Liang et al. (2017), ORSV and CMV were selected for the specific detection of the CymMV detection system. In our research, CMV, TMV, CarMV, and LMoV were used for specific detection, as they are similar to CymMV, ORSV, or CymRSV in morphology, structure, or belonging to the same family or genus.

### 4.5. Sensitivity of the virus detection system

Hosseini et al. (2021) reported that the Cq value of 0.02–0.2 pg. was between 32 and 39, indicating that the sensitivity detection threshold was less than 0.2 pg. Eun et al. (2000) used TaqMan real-time RT–PCR to detect CymMV and ORSV at a sensitivity of 10^4^ copies or 5 fg. Moreover, Geng et al. (2020) reported that the sensitivity detection threshold of *bovine parvovirus* (BPV) and *bovine coronavirus* (BCoV) was 200 copies/μL, and the sensitivity detection threshold of *bovine parainfluenza virus* (BPIV) was 20 copies/μL. Cao et al. (2022) reported that the sensitivity detection threshold was 50 copies/assay, which was 10–100-fold that of RT–PCR. Liang et al. (2017) reported that the sensitivity detection threshold of CymMV was 2.66 × 10 copies/μL, which was 100-fold that of RT–PCR. The sensitivity detection threshold identified in our study was 1 copy to 10^2^ copies or 6.2 × 10^−3^ fg to 6.2 × 10^−1^ fg, 10–10^4^-fold that was noted for RT–PCR.

### 4.6. Repeatability of the virus detection system

Geng et al. (2020) reported that the coefficient of variation (CV) for intra-and interassay was less than 2%. Cao et al. (2022) reported that the CVs for intra-and interassay ranged between 0.68–1.86% and 0.85–3.19%, respectively. Liang et al. (2017) reported that the CVs for intra-and interassay were less than 2.58 and 2.45%, respectively. In this research, intra-and interassay CVs were less than 0.55 and 0.95%, respectively. The low CVs represent the good repetition of the same concentration across three replicates and three separate experiments, indicating the good stability of this experiment.

### 4.7. Verification and application of plant

Jia et al. (2004) simultaneously detected CMV, TMV, and *potato virus Y* in 4 lilies with specific gene chips. Hosseini et al. (2021) confirmed the presence of *D. longicolla*, *D. caulivora*, *D. eres*, and *D. novem* and healthy soybean stems and soybean seeds (including whole seeds, seed coats, and uncoated seeds). Geng et al. (2020) reported using stool samples and nasal swab samples from seven cattle farms for verification. Ali R. N. et al. (2014) assessed a total of 31 orchid samples from six different orchid genera (*Cymbidium*, *Brassia*, *Dendrobium*, *Phalaenopsis*, *Masdevallia*, and *Oncidium*) as verification of the system. Liang et al. (2017) assessed 12 *Phalaenopsis* samples in triplicate and calculated the average Cq value and the viral content copy number of CymMV. Our research detected 66 orchids from five different orchid genera (*P. amabilis*, *Dendrobium nobile*, *Zygopetalums*, *Haraella retrocalla*, and *Oncidium hybridum*) by triplex taqman qRT–PCR and gene chip for plant verification. The detection results demonstrated that the triplex taqman qRT–PCR detection rate is higher than gene chip detection, which means that the sensitivity of the established triplex taqman qRT-PCR was higher than the gene chip.

### 4.8. Triplex TaqMan qRT–PCR assessment of CymMV, ORSV, and CymRSV

With the current increase in the exchange frequency of global germplasm resources of *P. amabilis* and other orchids (Koh et al., 2014), the trend in virus infection is becoming more severe, and there is no effective agent for the treatment of viral diseases in orchids. Therefore, the early detection of viral diseases and early isolation and treatment of diseased individuals is essential (Guo et al., 2011; Huang et al., 2018). CymMV, ORSV, and CymRSV are widely distributed in *P. amabilis* and other orchids. This paper describes a rapid, accurate, and precise triplex qRT–PCR detection system for the simultaneous detection of the said viruses and demonstrates the successful application of this method in *Phalaenopsis*, *Zygopetalums*, *Oncidium hybridum,* and *Dendrobium* orchids.

We chose qRT–PCR for the assay as it permits sensitive and specific detection of pathogens, and the use of TaqMan probes with different fluorescent dyes also permits parallel detection of different viruses in multiplex qPCR reactions (Elnifro et al., 2000; Schena et al., 2004). Moreover, qPCR saves a considerable amount of time compared to RT-PCR and enables multiple target detection efficiently. Thus, multiplex qPCR achieves efficient virus detection. This method can save much money, especially in the context of large sample numbers (Hosseini et al., 2021). In contrast, multiplex assays are more challenging to design than uniplex reactions. In a multiplex system, probes and primers must be specific to just one virus; otherwise, cross-detection of different species occurs (Elnifro et al., 2000; Kim and Choi, 2015). The concentration of primers and probes can also affect the PCR amplification efficiency. Specifically, a low concentration can lead to an incomplete reaction, whereas a high concentration can inhibit the reaction (Brownie et al., 1997). In addition, the annealing temperature can affect the specificity and amplification efficiency of PCR (Brownie et al., 1997). Therefore, this study has optimized a design by investigating the effects of these three factors (annealing temperature, primer, and probe concentration) on the triple qRT–PCR assay.

In general, the E is acceptable between 90 and 110%. In our assay, the Es of the standard curves of the three plasmids are all within this range (99–101%) and exhibited a linear trend (*R*^2^ = 0.999), indicating that the experimental conditions are optimal (Brownie et al., 1997). Furthermore, the standard curves we established for the three viruses also reflected the actual pathogen load in the infected tissues, thus making it possible to quantify the virus content. The lowest sensitivity of this triplex assay is 1 copy/assay for all three plasmids, and the sensitivity values for CymMV, ORSV, and CymRSV detection are 10, 100, and 100 copies/assay, respectively. These values represent a 10-to 10^4^-fold increase compared to conventional PCR. The specificity amplification curves of triplex real-time PCR confirmed the specificity of the primer-probe sets by demonstrating that each primer-probe set amplified a single product for its respective target species. The accuracy of this multiplex assay was also evaluated using genes from other significant orchid virus strains and healthy leaves as negative controls; no false-positive results were observed. Viruses often infect leaves in various combinations and concentrations; consequently, we constructed a codetection assay. This method demonstrated adequate precision and sensitivity while detecting different combinations of these three viruses.

## 5. Conclusion

The TaqMan triplex assay established in this study exhibits high specificity, sensitivity, and repeatability for the simultaneous detection, quantitation, and differential detection of CymMV, ORSV, and CymRSV. This methodology can be employed to achieve early detection, isolation, and prevention of spread of the said viruses from poisoned seedlings/potted flowers to improve plant quality and economic benefits.

## Data availability statement

The original contributions presented in the study are included in the article/Supplementary material, further inquiries can be directed to the corresponding author.

## Author contributions

AS and LW established the triplex qRT–PCR assay and manuscript writing. YZ and XY collected and managed the plant materials. YW, DY, and WL were responsible for the collection of literature. LW and XW revised the manuscript, supervision, and funding acquisition. All authors contributed to the article and approved the submitted version.

## Funding

This study was supported by Major Science and Technology Special Program of Yunnan Province (202102AE090052) and by Yunnan Seed Industry Joint Laboratory Project (202205AR070001-05).

## Conflict of interest

The authors declare that the research was conducted in the absence of any commercial or financial relationships that could be construed as a potential conflict of interest.

## Publisher’s note

All claims expressed in this article are solely those of the authors and do not necessarily represent those of their affiliated organizations, or those of the publisher, the editors and the reviewers. Any product that may be evaluated in this article, or claim that may be made by its manufacturer, is not guaranteed or endorsed by the publisher.
