# Supplementary material for: Establishment of a triplex TaqMan quantitative real-time PCR assay for simultaneous detection of Cymbidium mosaic virus, Odontoglossum ringspot virus and Cymbidium ringspot virus
Source: Front Microbiol. 2023 May 19;14:1129259. doi: 10.3389/fmicb.2023.1129259 (PMC10235546; doi:10.3389/fmicb.2023.1129259)
Supplement: Supplementary file 1 [file Data_Sheet_1.docx]

Supplementary Material

Establishment of a Triplex TaqMan Quantitative Real-time PCR Assay for Simultaneous Detection of *Cymbidium Mosaic Virus*, *Odontoglossum Ringspot Virus* and *Cymbidium Ringspot Virus*

Aiqing Sun*

*** Correspondence:**

Lihua Wang: wanglihua2525@outlook.com

Xuewei Wu: wuxuewei@ynu.edu.cn

# Supplementary Figures


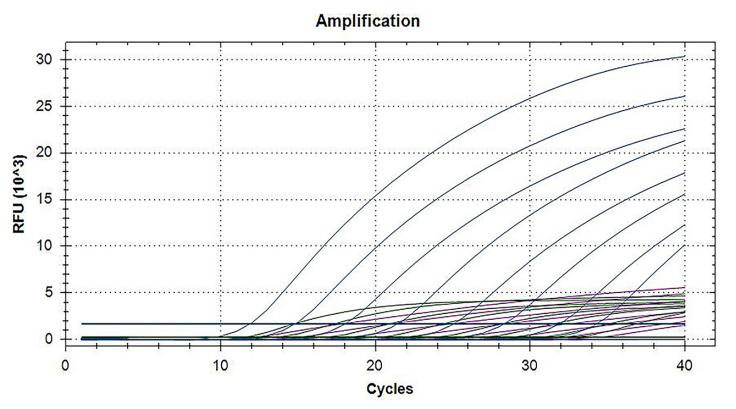

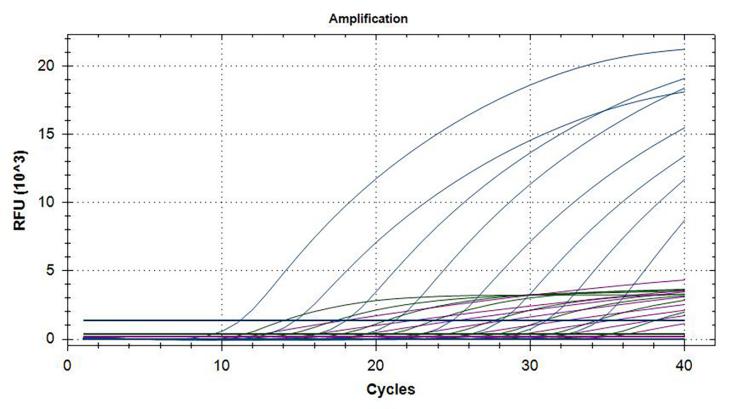


**A C**


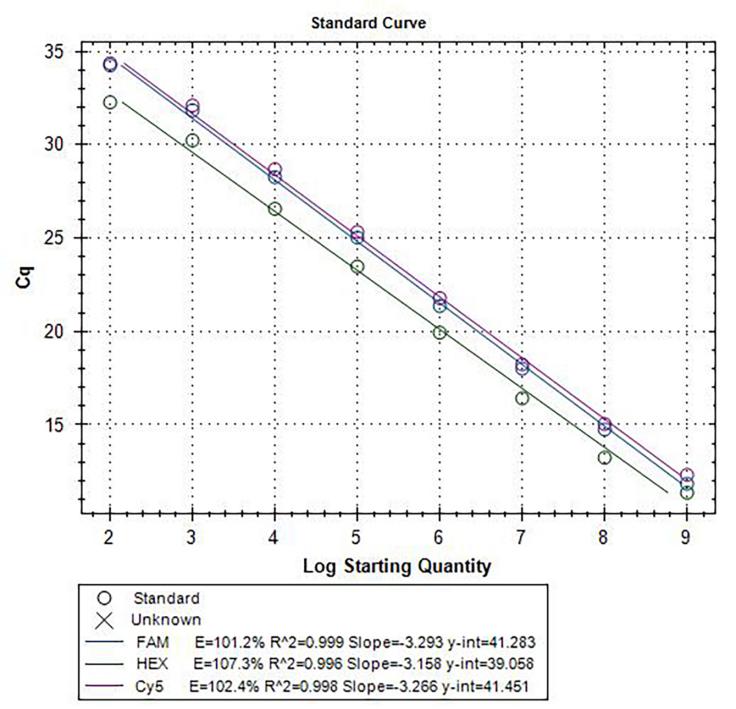

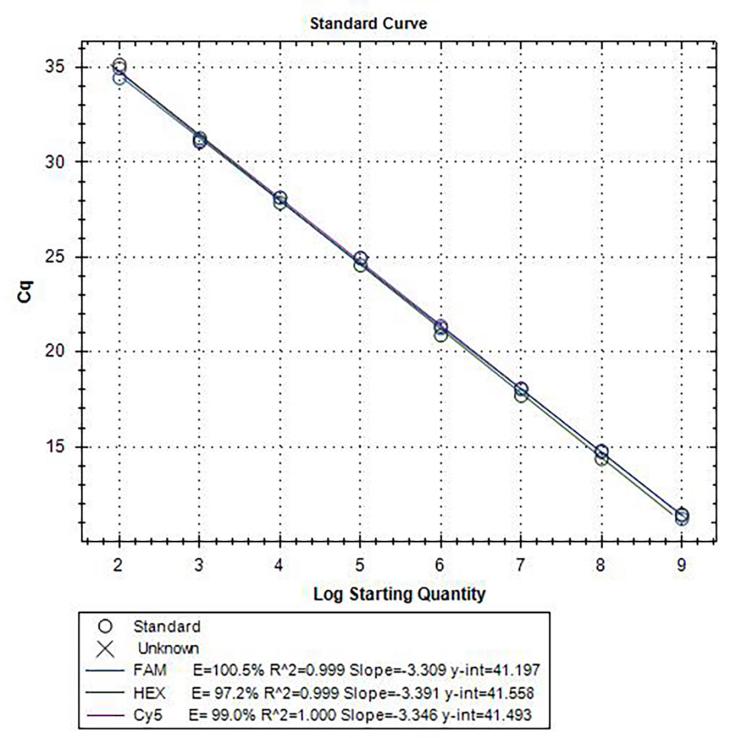


**B D**


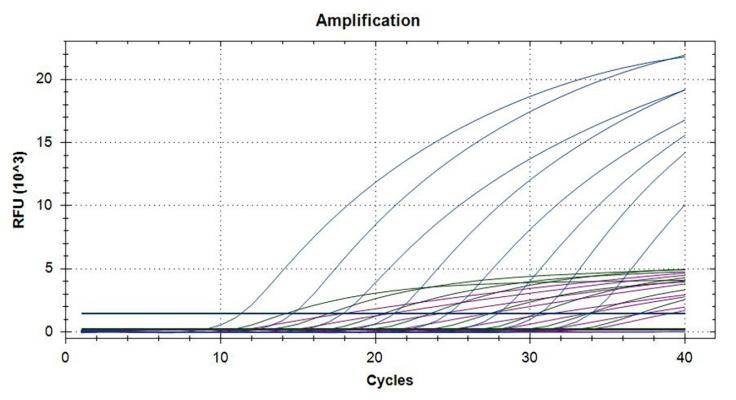

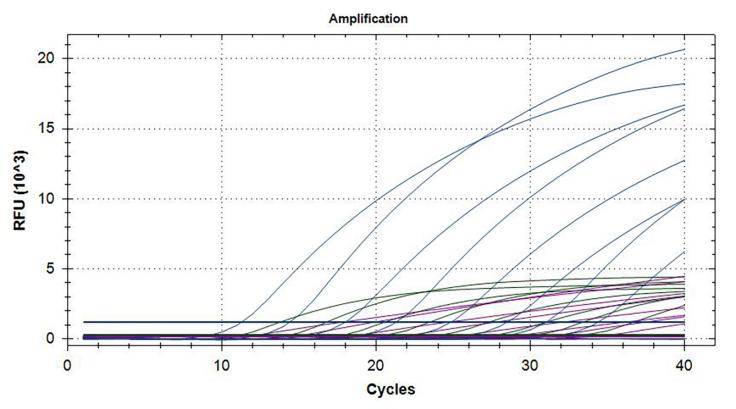


**E G**


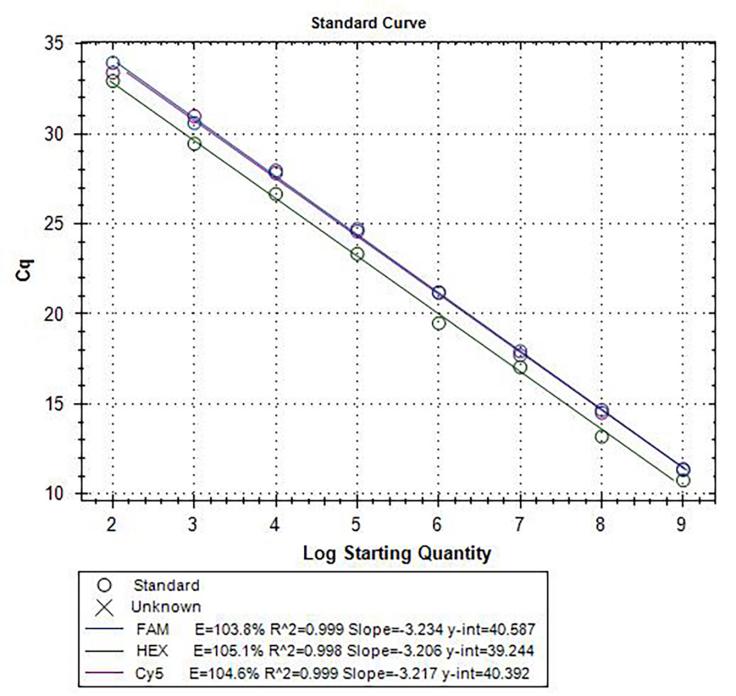

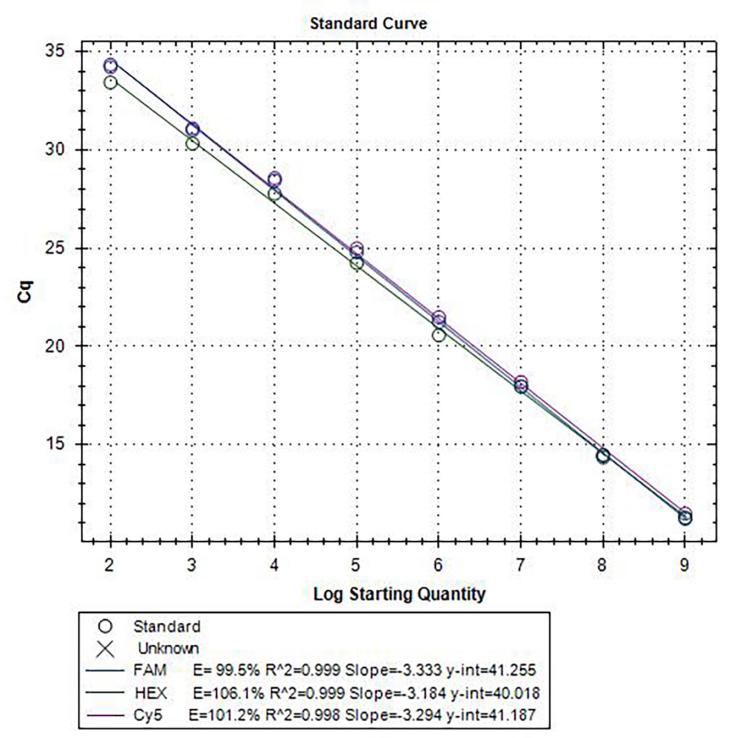


**F H**


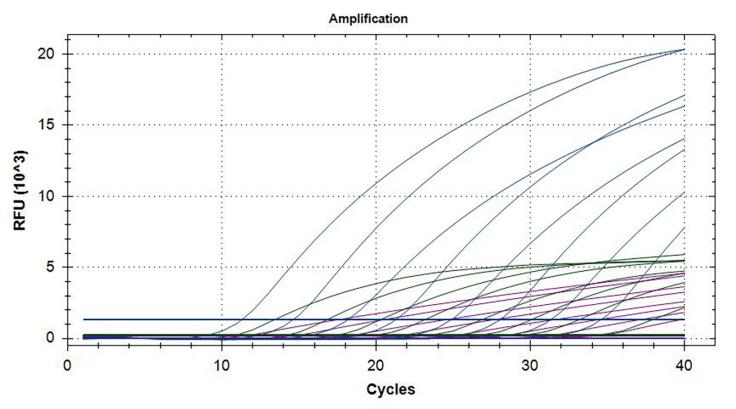


**I**


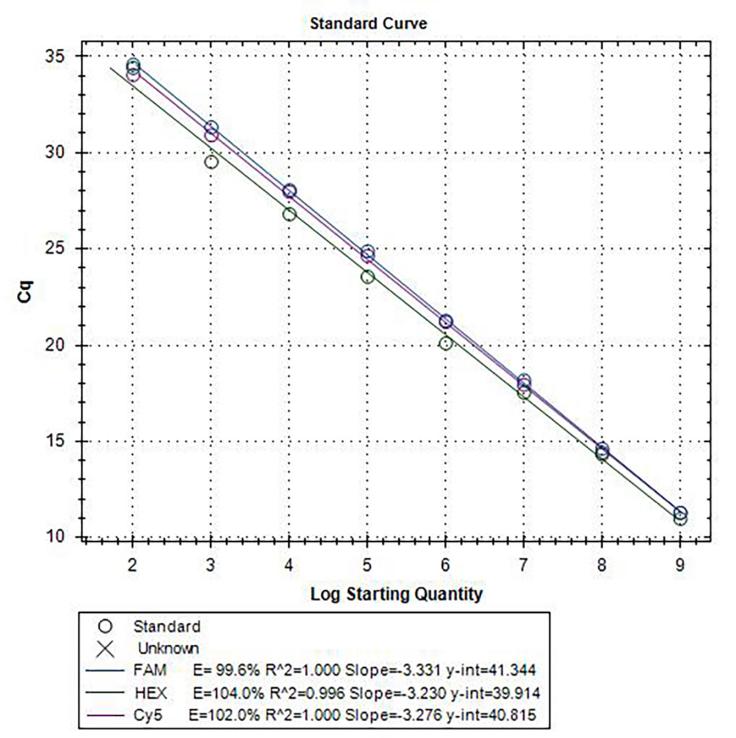


**J**

**Supplementary Figure 1.** The real-time PCR amplification curve and standard curve optimization results. **(A-B)** The amounts of CymMV, ORSV, and CymRSV primers was 0.5 μL and the probe was 1.0 μL, Tm=59 °C. **(C-D)** The amounts of CymMV, ORSV, and CymRSV primers was 0.5 μL and the probe was 1.0 μL, Tm=60 °C. **(E-F)** The primer and probe of CymMV was 0.5 μL and 1.0 μL respectively. The primers and probes of ORSV was 0.6 μL and 1.2 μL respectively, CymRSV is consistent with ORSV. Tm=59 °C. **(G-H)** The primer and probe of CymMV was 0.5 μL and 1.0 μL respectively. The primers and probes of ORSV and CymRSV were 0.6 μL and 1.2 μL respectively. Tm=60 °C. **(I-J)** The primer and probe of CymMV was 0.5 μL and 1.0 μL respectively. The primers and probes of ORSV was 0.7 μL and 1.4 μL respectively. The primers and probes of CymRSV was 0.6 μL and 1.2 μL respectively.

**
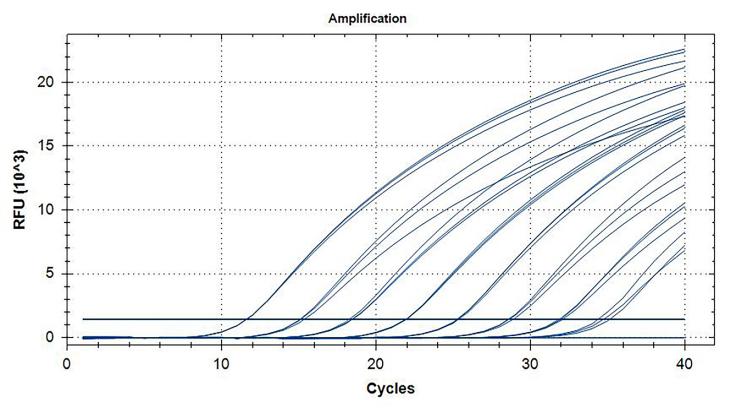
**

**A**

**
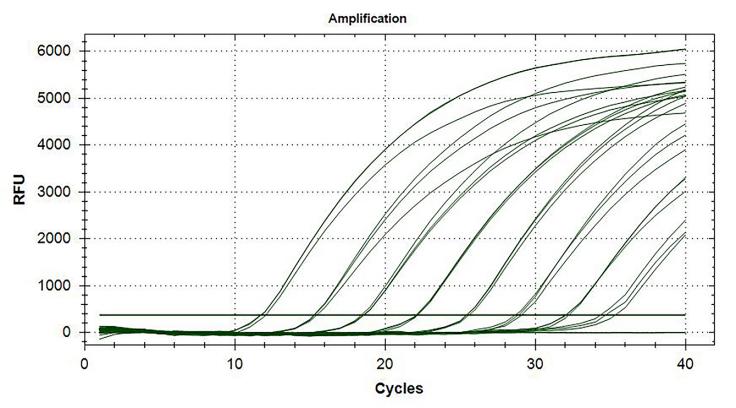

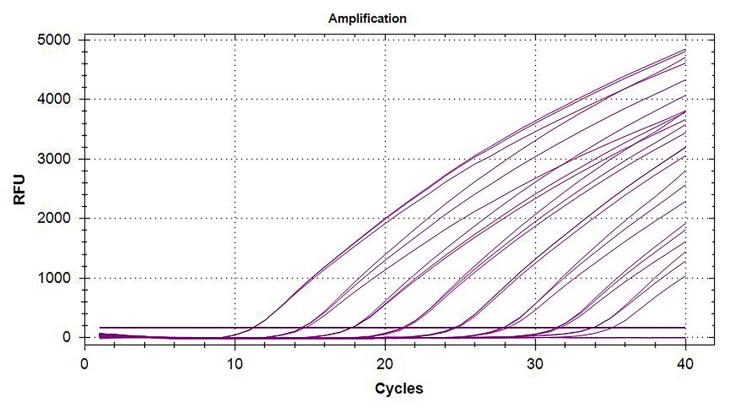
B C**

**Supplementary Figure 2.** Amplification curves and standard curves of the triplex assay. The mixed 10^9^ copies/assay to 10^2^ copies/assay plasmid was used as the template for qRT‒PCR. Amplification curves of the triplex assay for the detection of FAM channel (CymMV) **(A)**, HEX channel (ORSV) **(B)**, and Cy5 channel (CymRSV) **(C)**.
